# Prevention of Hypomineralization In Auditory Ossicles of Vitamin D Receptor (Vdr) Deficient Mice

**DOI:** 10.3389/fendo.2022.901265

**Published:** 2022-06-06

**Authors:** Maximilian M. Delsmann, Jonathan Peichl, Timur A. Yorgan, Frank Timo Beil, Michael Amling, Marie B. Demay, Tim Rolvien

**Affiliations:** ^1^Department of Trauma and Orthopaedic Surgery, Division of Orthopaedics, University Medical Center Hamburg-Eppendorf, Hamburg, Germany; ^2^Department of Osteology and Biomechanics, University Medical Center Hamburg Eppendorf, Hamburg, Germany; ^3^Department of Otorhinolaryngology, University Medical Center Hamburg-Eppendorf, Hamburg, Germany; ^4^Endocrine Unit, Massachusetts General Hospital, Harvard Medical School, Boston, MA, United States

**Keywords:** vitamin D receptor, mineralization, qBEI, auditory ossicles, hearing

## Abstract

Intact mineralization of the auditory ossicles - the smallest bones in the body - is essential for sound transmission in the middle ear, while ossicular hypomineralization is associated with conductive hearing loss. Here, we performed a high-resolution analysis of the ossicles in vitamin D receptor deficient mice (*Vdr^-/-^*), which are characterized by hypocalcemia and skeletal mineralization defects, and investigated whether local hypomineralization can be prevented by feeding a calcium-rich rescue diet (*Vdr^-/- res^*). In *Vdr^-/-^* mice fed a regular diet (*Vdr^-/- reg^*), quantitative backscattered electron imaging (qBEI) revealed an increased void volume (porosity, p<0.0001) along with lower mean calcium content (CaMean, p=0.0008) and higher heterogeneity of mineralization (CaWidth, p=0.003) compared to WT mice. Furthermore, a higher osteoid volume per bone volume (OV/BV; p=0.0002) and a higher osteocyte lacunar area (Lc.Ar; p=0.01) were found in histomorphometric analysis in *Vdr^-/- reg^* mice. In *Vdr^-/- res^* mice, full rescue of OV/BV and Lc.Ar (both p>0.05 vs. WT) and partial rescue of porosity and CaWidth (p=0.02 and p=0.04 vs. WT) were observed. Compared with *Hyp* mice, a model of X-linked hypophosphatemic rickets, *Vdr^-/- reg^* mice showed a lower osteoid volume in the ossicles (p=0.0002), but similar values in the lumbar spine. These results are consistent with later postnatal impairment of mineral homeostasis in *Vdr^-/-^* mice than in *Hyp* mice, underscoring the importance of intact mineral homeostasis for ossicle mineralization during development. In conclusion, we revealed a distinct phenotype of hypomineralization in the auditory ossicles of *Vdr^-/-^* mice that can be partially prevented by a rescue diet. Since a positive effect of a calcium-rich diet on ossicular mineralization was demonstrated, our results open new treatment strategies for conductive hearing loss. Future studies should investigate the impact of improved ossicular mineralization on hearing function.

## Introduction

The vitamin D receptor (Vdr) is a calcitriol (1,25-dihydroxyvitamin D_3_) binding nuclear receptor encoded by the *Vdr* gene that regulates gene transcription by binding to a vitamin D response element in the promoter region of certain genes (1). The *Vdr* knockout mouse (*Vdr^-/-^*), a model of hereditary vitamin D-dependent rickets type II, lacks vitamin D-dependent gene expression, resulting in impaired mineral ion homeostasis and related defects like rickets (2, 3). In the first 18 days of life, a non-saturable 1,25-dihydroxyvitamin D-independent mechanism maintains calcium absorption (4). After weaning, secondary hyperparathyroidism compensates for the reduced intestinal calcium absorption by PTH-mediated bone resorption; however, the serum calcium level drops after about 4 weeks of age (3, 5). The calcium level stabilizes 30% below that of wildtype (WT) mice, while phosphate is increasingly eliminated *via* the kidney due to secondary hyperparathyroidism, resulting in hypophosphatemia (3, 5).

Altered calcium and phosphate metabolism lead to marked skeletal abnormalities such as severe osteomalacia with a 30-fold greater osteoid volume compared to control littermates, leading to a significant reduction of biomechanical properties and increased bone fragility (6, 7). Furthermore, the growth plates present a marked disorganization and a significant increase in the length of the hypertrophic chondrocyte layer (5, 6, 8). In *Vdr^-/-^* mice fed a calcium-/phosphate-rich rescue diet from 16 days of age, normalized mineral ion homeostasis was observed with normal osteoid and tibial bone volume, showing that intestinal calcium absorption is a critical factor in mineral metabolism and bone mineralization (6, 8).

Poor bone mineralization (i.e., rickets, osteomalacia) is primarily characterized by skeletal complications such as insufficiency fractures. Nonetheless, extraskeletal complications such as dental problems or hearing loss are also recognized. In the latter context, we and others have previously demonstrated that the integrity and especially bone mineralization of auditory ossicles is of decisive importance for sound transmission in the hearing process (9–12). However, it remains unknown whether dietary or bone-targeted treatments ameliorate ossicular hypomineralization and conductive hearing loss. This question remains particularly worthy of investigation considering that in humans and mice the development and mineralization of ossicles is normally completed shortly after birth and little ossicular remodeling accompanied by high matrix mineralization has been observed across species (10, 13, 14). Therefore, this study aims to characterize the mineralization properties of the auditory ossicles in *Vdr^-/-^* mice, also focusing on the treatment effects by a calcium-/phosphate-rich diet.

## Materials and Methods

### Animals and Experimental Design

*Vdr*-deficient mice (B6.129S4-*Vdr*^tm1Mbd^/J) on regular (*Vdr^-/- reg^*) or calcium-/phosphate-rich rescue diet (*Vdr^-/- res^*) and their wild-type (WT) littermates were included from a previous study for high-resolution skeletal analysis of auditory ossicles (7). All mice were on a C57BL/6J background, maintained in a specific pathogen-free environment with a 12-h light/dark cycle, 45–65% relative humidity, and 20–24°C ambient temperature in open cages with wood shavings bedding and nesting material. *Vdr^-/- reg^* and WT mice were fed autoclaved Purina rodent chow containing 1% calcium, 0.67% phosphate, 0% lactose, and 4.4 IU vitamin D/g (regular diet). To normalize mineral ion levels, *Vdr^-/- res^* mice were fed γ-irradiated rescue chow (TD96348, Teklad, Madison, WI) containing 2% calcium, 1.25% phosphate, and 20% lactose with 2.2 IU vitamin D/g bodyweight. Both diets were initiated after weaning on day 16. For each genotype, five mice were analyzed at 10 weeks of age. Only male mice were examined. For comparative purposes, we also analyzed the auditory ossicles and lumbar vertebral bodies of four age-matched male *Hyp* mice (B6.Cg-*Phex*^Hyp^/J, Jackson Laboratory, #000528, C57BL/6J background), a model of X-linked hypophosphatemic rickets obtained in the context of a previous study (15). All animal preparations were approved by the “Behörde für Umwelt und Gesundheit der Hansestadt Hamburg” (Org529, G14/68).

### Sample Preparation and Quantitative Backscattered Electron Imaging

Preparation of middle ears and isolation of auditory ossicles were performed under a stereomicroscope. All isolated specimens were fixed in 3.7% formaldehyde, dehydrated in an ascending ethanol series, and embedded undecalcified in methyl methacrylate. To analyze the bone mineral density distribution (BMDD), the embedded auditory ossicles (malleus and stapes) were polished to a coplanar finish, carbon coated, and subsequently analyzed by quantitative backscattered electron imaging (qBEI), consisting of a scanning electron microscope (LEO 435 VP, LEO Electron Microscopy Ltd.; Cambridge, England) with a backscattered electron detector (Type 202; K.E. Developments Ltd.; Cambridge, UK). Polishing was performed using a surface grinding machine (EXAKT 400 CS, EXAKT, Norderstedt, Germany). Initially, the 1200 grit silicon carbide wet sandpaper (Allied High Tech Products Inc., Rancho Dominguez, USA) was used for grinding. The exact grinding duration was adjusted to each specimen to obtain an appropriate cross-section of the specimen. After visual confirmation of the optimal cross-section, the specimen was now polished for 4 minutes using 4000 grit silicon carbide wet sandpaper. The scanning electron microscope was operated at 20 kV and 680 pA at a constant working distance, as described previously (16, 17). Images were taken at 100x magnification, representing a pixel size of 1.15 µm. The generated gray values represent the mean calcium content (mean Ca-wt%) of the cross-sectioned bone (18). Brightness and contrast of the qBEI images were calibrated using carbon and aluminum standards: The gray values assigned to carbon and aluminum were 4.8 and 222, respectively. Image analysis was performed using ImageJ (ImageJ 1.42, National Institutes of Health, Bethesda, USA) (19) and a custom MATLAB-based program (TheMathWorks, Inc., Natick, USA). Gray values proportional to calcium content were used to determine the mean calcium content (CaMean, wt%), standard deviation (i.e., heterogeneity of mineralization, CaWidth, wt%), and most frequent calcium content (CaPeak, wt%). Furthermore, the void volume (i.e., porosity) as well as the osteocyte lacunar number (N.Ot.Lc/B.Ar, 1/mm^2^) and area (Lc.Ar, μm^2^) were assessed. Following standardized thresholding using ImageJ, black voids within the mineralized bone with a size threshold of 4-100 µm^2^ were classified as osteocyte lacunae while voids larger than 100 µm^2^ were classified as porosity.

### Histology and Histomorphometry

The samples were cut into 4 µm sections using a rotation microtome (CVT 4060E, microTec, Walldorf, Germany) and subsequently stained with von Kossa-van Gieson and toluidine blue according to previously described protocols (20). Histomorphometric analysis was performed according to ASBMR guidelines (21) using a bright- field light microscope (Axioskop 40, Carl Zeiss Vision GmbH, Germany) equipped with Osteomeasure Software (OsteoMetrics Inc., Atlanta, USA). The bone volume per tissue volume (BV/TV, %) and the osteoid volume per bone volume (OV/BV, %) were analyzed during histological analysis.

### Statistical Analysis

Statistical analyses were performed using GraphPad Prism (version 9.0, GraphPad Software, La Jolla, USA). Continuous variables are expressed as absolute values or the mean ± standard deviation (SD). Shapiro–Wilk test was used to evaluate the normal distribution of the data. For comparison of normally distributed data among the three groups, one-way ANOVA and repeated measures with Holm-Sidak correction was applied, and for non- parametric data, the Kruskal–Wallis test with Dunn’s multiple comparison test was performed. For comparison between two groups, Student’s t-test was used for normally distributed data and Mann-Whitney U-test for nonparametric data. The level of significance was defined as p<0.05.

## Results

### Partial Rescue of Hypomineralization in the Auditory Ossicles of Vdr^-/-^ Mice

**Figure 1:**
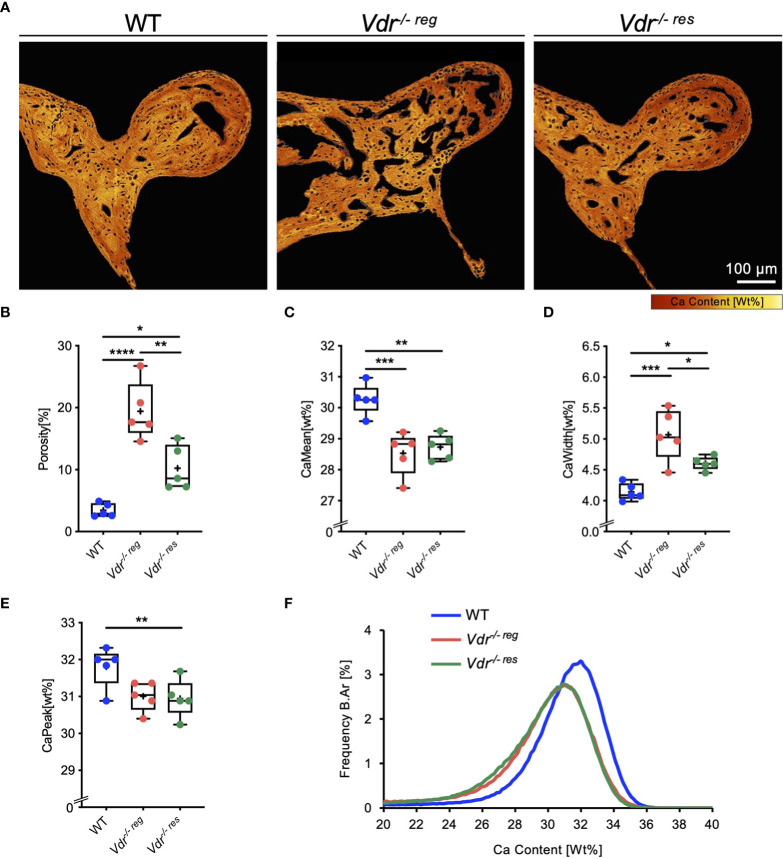
Partial reversibility of porosity and bone mineral density distribution (BMDD) in the malleus evaluated by qBEI. **(A)** Representative, pseudo-colored images of the qBEI analysis of the orbicular apophysis of the malleus in WT, *Vdr^-/- reg^* and *Vdr^-/- res^* mice. The evaluation was based on the quantification of the **(B)** porosity, **(C)** mean calcium content (CaMean), **(D)** mineralization heterogeneity (CaWidth), and **(E)** peak of the calcium distribution (CaPeak). **(F)** Bone mineral density distribution (BMDD) histograms of the malleus of WT (Blue curve), *Vdr^-/- reg^* (red curve), *Vdr^-/- res^* mice (green curve). ANOVA and repeated measures with Holm-Sidak correction was performed in panels B-E. *p<0.05; **p<0.01, ***p<0.001, ****p<0.0001.

High-resolution imaging of the malleus by qBEI (**Figure 1A**) indicated markedly impaired bone mineralization as well as higher porosity in *Vdr^-/- reg^* mice compared to WT mice (19.42 ± 4.65% *vs.* 3.48 ± 1.09%; p<0.0001; **Figure 1B**). In *Vdr^-/- res^* mice, the porosity (10.23 ± 3.61%) was significantly lower than in *Vdr^-/- reg^* mice (p=0.003) but remained significantly higher than in WT mice (p=0.02). The mean calcium content (CaMean) was significantly higher in WT mice (30.27 ± 0.50%; **Figure 1C**) compared to both *Vdr^-/- reg^* (28.53 ± 0.70%; p=0.0008) and *Vdr^-/- res^* mice (28.73 ± 0.41%; p=0.002), with similar values in *Vdr^-/- reg^* and *Vdr^-/- res^* mice (p=0.83). However, significantly lower mineralization heterogeneity was detected in *Vdr^-/- res^* than in *Vdr^-/- reg^* mice (CaWidth, 4.61 ± 0.11% *vs.* 5.07 ± 4.16%; p=0.04; **Figure 1D**). The highest mineralization homogeneity was found in WT mice (4.14 ± 0.14%), significantly differing from *Vdr^-/- reg^* (p=0.003) and *Vdr^-/- res^* mice (p=0.04). The CaPeak remained lower in *Vdr^-/- res^* compared to WT mice (30.94 ± 0.51% *vs.* 31.81 ± 0.55%; p=0.04; **Figure 1E**). BMDD histograms indicated a rightward shift and a narrower calcium distribution curve in WT compared to both *Vdr^-/- reg^* and *Vdr^-/- res^* mice (**Figure 1F**), reflecting the overall higher and more homogeneous mineralization.

**Figure 2:**
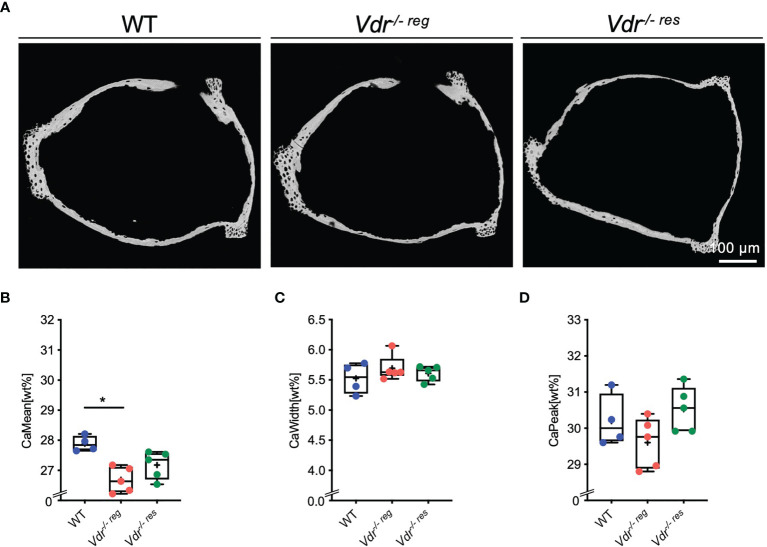
BMDD characteristics of the stapes analyzed by qBEI. **(A)** Representative images of qBEI analysis of the stapes in WT, *Vdr^-/- reg^* and *Vdr^-/- res^* mice. Mineralization characteristics were evaluated by analyzing the **(B)** mean calcium content (CaMean), **(C)** mineralization heterogeneity (CaWidth) and **(D)** peak of the calcium distribution (CaPeak). ANOVA and repeated measures with Holm-Sidak correction was performed in panels **(B–D)**. *p<0.05.

In the stapes (**Figure 2A**), qBEI revealed a significantly lower CaMean in *Vdr^-/- reg^* (26.69 ± 0.42%) than the WT littermates (27.89 ± 0.26%; p=0.003; **Figure 2B**), with no differences in CaMean between WT and *Vdr^-/- res^* mice (27.18 ± 0.46%, p=0.17). Further, no differences between the groups were observed in CaWidth (**Figure 2C**) and CaPeak (**Figure 2D**).

### Prevention of Hyperosteoidosis in the Malleus and Comparison With Vertebrae

**Figure 3:**
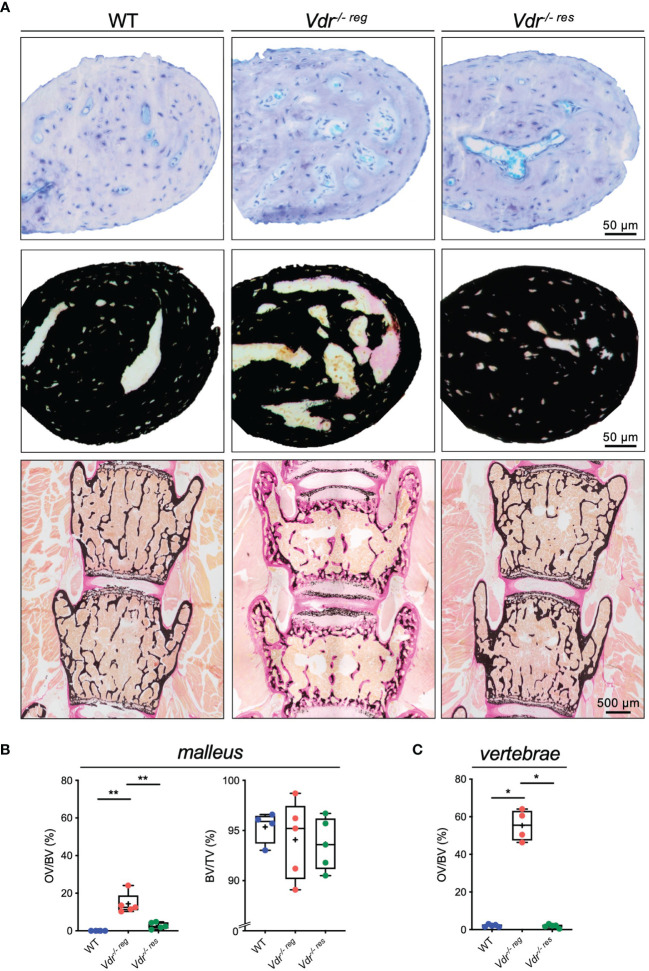
Undecalcified histology identifies partial reversibility of hyperosteoidosis in the malleus as opposed to the lumbar spine. **(A)** Representative histological images of toluidine blue (upper panel) and von Kossa-van Gieson (middle panel) stained orbicular apophysis of the malleus in 10-weeks old WT, *Vdr^-/- reg^* and *Vdr^-/- res^* mice. Histological images of the lumbar vertebral bodies in von Kossa-van Gieson staining (lower panel). **(B)** Histomorphometric evaluation including osteoid volume per bone volume (OV/BV) and bone volume fraction (BV/TV) in the malleus and **(C)** OV/BV in the vertebral body. ANOVA and repeated measures with Holm-Sidak correction was performed in panels **(B–C)**. *p<0.05; **p<0.01.

Histological analysis of the malleus (**Figure 3A**) revealed full reversibility of the strongly increased osteoid levels in *Vdr^-/- res^* compared to *Vdr^-/- reg^* mice. Specifically, the malleus in *Vdr^-/- reg^* mice presented a significantly higher OV/BV compared to WT mice (14.38 ± 5.56% *vs.* 0.0 ± 0.0%; p=0.0002) and to *Vdr^-/- res^* mice (2.78 ± 1.74%; p=0.0008). BV/TV was equal in all groups (**Figure 3B**). In comparison, the vertebral bodies of *Vdr^-/- reg^* mice also exhibited a significantly higher OV/BV compared to WT mice (55.3 ± 8.32% *vs.* 2.1 ± 0.67%; p<0.0001; **Figure 3C**), but OV/BV was fully corrected in *Vdr^-/- res^* mice with significantly lower values than in *Vdr^-/- reg^* mice (1.88 ± 0.99%; p<0.0001) but without differences compared to WT mice (p>0.05).

### Full Rescue of Osteocyte Lacunar Enlargement

**Figure 4:**
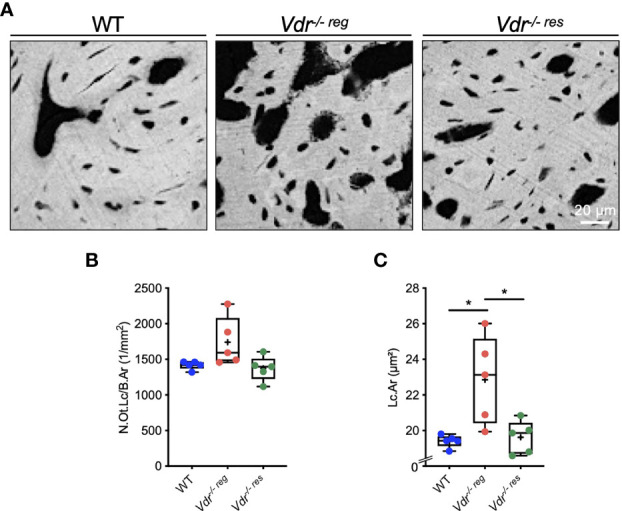
Osteocyte lacunar characteristics of the malleus analyzed by qBEI. **(A)** Representative qBEI images showing osteocyte lacunae in the malleus of WT, *Vdr^-/- reg^* and *Vdr^-/- res^* mice. **(B)** Quantification of the osteocyte lacunar number (N.Ot.Lc/B.Ar) and **(C)** mean osteocyte lacunar area (Lc.Ar). ANOVA and repeated measures with Holm-Sidak correction was performed in panels **(B)** and **(C)** *p<0.05.

Since osteocytes are known to mediate bone remodeling and bone mineralization, and the osteocyte’s function is highly influenced by its morphology, we next analyzed the osteocytes’ lacunar characteristics by qBEI (**Figure 4A**). We found no significant differences regarding the number of osteocyte lacunae (N.Ot.Lc/B.Ar, p>0.05 for all comparisons) (**Figure 4B**). Nonetheless, evaluating the lacunar area (Lc.Ar), *Vdr^-/- reg^* mice (22.84 ± 2.48 µm^2^) exhibited a significantly higher osteocyte lacunar area compared to WT mice (19.41 ± 0.35 µm^2^; p=0.01), while *Vdr^-/- res^* mice (19.62 ± 0.93 µm^2^) showed a full rescue in Lc.Ar (p=0.02) (**Figure 4C**).

### Peculiarities of Ossicular Mineralization Demonstrated by Comparative Analysis of Hyp Mice

**Figure 5:**
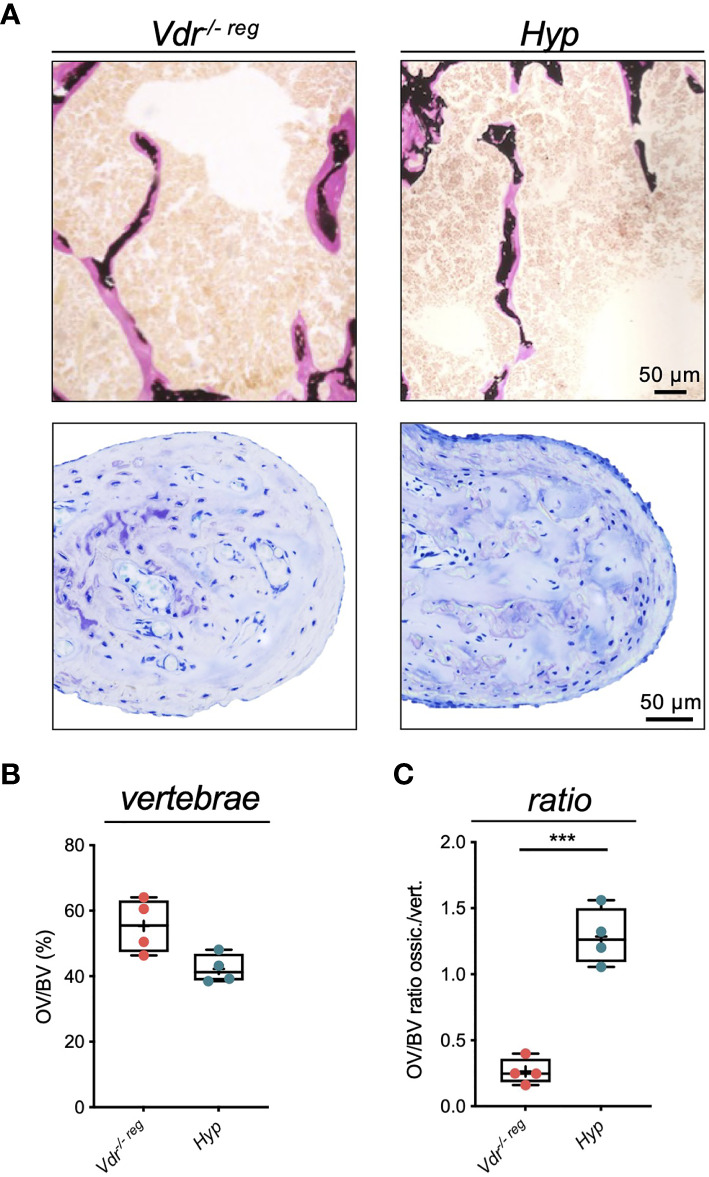
Comparative analysis of osteoid accumulation in the lumbar spine and malleus in *Vdr^-/- reg^* and *Hyp* mice reflects peculiarities in ossicular mineralization. **(A)** Representative images of bone trabeculae in the vertebral bodies (von Kossa-van Gieson staining, upper panel) and of the malleus (toluidine blue staining, lower panel) in *Vdr^-/- reg^* mice compared to *Hyp* mice. Osteoid is stained pink (von Kossa-van Gieson) or light blue (toluidine blue) **(B)** Quantification of osteoid volume per bone volume (OV/BV) in the vertebrae. **(C)** OV/BV ratio between malleus and vertebrae. Student’s t-test was used in panels **(B)** and **(C)** ***p<0.001.

Comparing the osteoid levels of *Vdr^-/- reg^* and *Hyp* mice in the lumbar spine (**Figure 5A**), a non-significantly higher OV/BV was observed in the vertebral bodies of *Vdr^-/- reg^* compared to *Hyp* mice (55.30 ± 8.32% *vs.* 42.25 ± 4.42%; p=0.06) (**Figure 5B**). In the malleus, this pattern was reversed with a markedly lower amount of osteoid in *Vdr^-/- reg^* compared to *Hyp* mice, reflected by a significantly higher OV/BV ratio between ossicles and vertebrae in *Hyp* mice (1.29 ± 0.21 *vs.* 0.26 ± 0.10; p=0.0001) (**Figure 5C**).

## Discussion

In this study, we aimed to investigate the micro-morphological characteristics of the auditory ossicles in *Vdr^-/-^* mice, focusing on the bone mineral density distribution and its changes by feeding a calcium-/phosphate-rich diet. In *Vdr^-/- reg^* mice, qBEI revealed significantly increased porosity of the malleus along with a reduced and more heterogeneous mineralization, while histological analysis showed increased osteoid volume and higher osteocyte lacunar area compared to WT littermates. In *Vdr^-/- res^* mice, complete correction of osteoid volume and lacunar area and partial correction of porosity and mineralization heterogeneity could be observed in the malleus, while the mean calcium content remained unchanged. Overall, the calcium-/phosphate-rich diet resulted in distinct improvements of the pre-existing ossicular hypomineralization towards physiological conditions, demonstrating for the first time that a bone-targeted diet improves the bone quality of auditory ossicles.

Interestingly, the osteoid volume in the auditory ossicles of *Vdr^-/- reg^* mice was considerably lower than in the lumbar spine, while the rescue effect appeared to be relatively attenuated. This phenomenon is likely due to a combination of rapid postnatal ossicular development with little remodeling after the first few postnatal weeks (10), and unaffected mineral homeostasis until the third week in *Vdr^-/-^* mice (3). Specifically, in the auditory ossicles, bone development is completed more rapidly than in other bones (10, 22, 23). The longitudinal growth is already completed after 20 days and the endochondral ossification between 6-12 weeks after birth (10, 22). During endochondral ossification, large capillary loops lined with endothelial cells can be seen in the ossicles immediately after birth, which becomes mineralized by specific type I and type II collagen-producing auditory osteoblasts (24, 25). Impaired mineral homeostasis may prevent unmineralized bone (i.e., osteoid) from undergoing regular mineralization. However, since impaired mineral homeostasis in *Vdr^-/-^* mice manifests not after weaning on postnatal day 16 (3, 5, 6), a large proportion of the mineralization process in the auditory ossicles may already be completed. Furthermore, in both mice and humans, rapid development is usually followed by a markedly lower bone remodeling rate in the ossicles compared to other bones (13). Therefore, the remineralization of the auditory ossicles in *Vdr^-/- res^* mice likely does not occur to the same extent as in the remaining skeleton. Together, considering the early completion of bone development and the overall low bone remodeling rate in the ossicles, the observed differences in mineralization in the ossicles compared to the spine of *Vdr^-/-^* mice might be explained.

Further evidence of the unique mineralization mechanisms of the ossicles is derived from a comparative analysis of *Hyp* mice. *Vdr^-/- reg^* mice presented a similar amount of osteoid in the lumbar spine but significantly lower amounts in the malleus compared with *Hyp* mice. In this regard, it is important to consider that the mineralization defect in *Hyp* mice manifests immediately postnatally, due to an early onset of impaired mineral homeostasis caused by increased renal phosphate wasting (26). Therefore, the mineralization processes of the ossicles in the first days of life cannot proceed in a regular manner in the *Hyp* mice, whereas they still take place normally in the *Vdr^-/- reg^* mice during the weaning period (3, 5, 6), resulting in a more sufficient mineralization of the ossicles in *Vdr^-/- reg^* mice. These results are contrasted by the findings in the spine, where development and mineralization take place beyond day 16, which is why vertebral hypomineralization is as severely affected in *Vdr*^-/-^ mice as in *Hyp* mice.

Regarding osteocyte lacunar characteristics, it is noteworthy that a full correction of increased lacunar area in the malleus was noted in *Vdr^-/- res^* compared to *Vdr^-/- reg^* mice. These results support the concept of osteocyte perilacunar remodeling (i.e., osteocytic osteolysis) previously observed in other bones of *Vdr^-/-^* mice, *Hyp* mice (27), and particularly in lactating mice (28). Although perilacunar remodeling was not further evaluated in this study, the complete correction of osteocyte lacunar area detected here argues for the dynamic role of osteocytes in the process of controlling matrix mineralization (29). In the context of auditory function, osteocytes could thus be assigned an indirect role *via* their control of matrix mineralization. The full correction is contrary to the low remodeling rates and argues for enough viability to ensure remineralization.

To interpret the clinical relevance of our findings in the context of hearing function, it is useful to acknowledge the auditory ossicles as a dynamic, functional unit, which is essential for sound transmission during the hearing process. In addition to pure sound transmission, the auditory ossicles play a crucial role in acoustic impedance matching and amplifying sound to ensure the transmission from the air-filled middle ear to the fluid-filled inner ear (i.e., cochlea) (30). Since a significant correlation between hearing capacity and the bone mineral density has been reported in humans (31), the physiological bone composition of the ossicles appears to provide an optimal mix of stability and elasticity that ensures functionality. In this context, it is also interesting that a high prevalence of hearing loss has been reported in a variety of genetic bone diseases, including X-linked hypophosphatemic rickets (XLH) and osteogenesis imperfecta (32, 33). Our findings are also relevant to the clinical observation that vitamin D deficiency has been identified as a risk factor for hearing loss in both children and the elderly (34, 35), with hypocalcemia being an additional independent risk factor in children (34).

Hearing loss has also been demonstrated in mouse models that recapitulate genetic bone diseases, such as in osteoprotegerin (Opg)-deficient mice (*Opg^-/-^)*, a decoy receptor for receptor activator of nuclear factor κ-β-ligand (RANKL), which is associated with markedly activated osteoclast activity (12). Normalization of resorption activity in *Opg^-/-^* mice with bisphosphonate therapy resulted in an improvement in hearing (9). A correlation between poor bone quality and hearing ability was also shown for other mouse models. In FGF23-deficient mice suffering from hyperphosphatemia and hypercalcemia with resulting defective bone mineralization (36), a mixed conductive and sensorineural hearing loss was reported (11), whereas in *Hyp* mice with FGF23 overexpression and consecutive hypophosphatemia and mineralization impairment, a predominant conductive hearing loss was present (10). In *Vdr^-/-^* mice, sensorineural hearing loss associated with loss of spiral ganglion cells in the basal turn has been previously reported, however, conductive hearing loss had not been investigated in this model (37).

The limitations of our study include the relatively small sample size and the fact that we did not perform hearing tests in *Vdr^-/-^* mice. Nonetheless, it seems likely that *Vdr^-/-^* mice exhibit conductive hearing loss due to hypomineralization of auditory ossicles. While we evidenced the beneficial effects of a therapeutic intervention on ossicular mineralization, the question whether conductive hearing loss can be counteracted by a specific diet or bone-targeted treatments needs to be investigated in future studies.

In conclusion, we here demonstrated a distinct mineralization defect in the auditory ossicles of *Vdr^-/-^* mice, which was partially reversed by a calcium-/phosphate-rich rescue diet. Since adequate mineralization in the middle ear is associated with functional sound conduction, the positive effects of a calcium-/phosphate-rich diet on ossicular mineralization open new treatment strategies for conductive hearing loss, which is commonly observed in patients with genetic bone diseases. Our results further highlight the importance of adequate mineral supply during early postnatal development to ensure sufficient ossicle quality.

## Data Availability Statement

The original contributions presented in the study are included in the article. Further inquiries can be directed to the corresponding author.

## Ethics Statement

The animal study was reviewed and approved by Behörde für Umwelt und Gesundheit der Hansestadt Hamburg (Org529, G14/68).

## Author Contributions

MMD: Data curation, Conceptualization, Visualization, Writing – original draft, Writing – review and editing. JP: Data curation, Writing – review & editing. TY: Data curation, Writing – review and editing. FB: Conceptualization, Writing – review & editing. MA: Conceptualization, Writing – review and editing. MBD: Conceptualization, Visualization, Writing – review and editing. TR: Data curation, Conceptualization, Visualization, Writing – original draft, Writing – review and editing. All authors contributed to the article and approved the submitted version.

## Conflict of Interest

The authors declare that the research was conducted in the absence of any commercial or financial relationships that could be construed as a potential conflict of interest.

## Publisher’s Note

All claims expressed in this article are solely those of the authors and do not necessarily represent those of their affiliated organizations, or those of the publisher, the editors and the reviewers. Any product that may be evaluated in this article, or claim that may be made by its manufacturer, is not guaranteed or endorsed by the publisher.
